# A rational experimental approach to identify correctly the working voltage window of aqueous-based supercapacitors

**DOI:** 10.1038/s41598-020-75851-7

**Published:** 2020-11-05

**Authors:** Willian G. Nunes, Bruno G. A. Freitas, Renato M. Beraldo, Rubens Maciel Filho, Leonardo M. Da Silva, Hudson Zanin

**Affiliations:** 1 Advanced Energy Storage Division, Center for Innovation on New Energies, Carbon Sci-Tech Labs and Manufacturing Group, School of Electrical and Computer Engineering, University of Campinas, Av. Albert Einstein 400, Campinas, SP 13083-852 Brazil; 2 Advanced Energy Storage Division, Center for Innovation on New Energies, School of Chemical Engineering, University of Campinas, Av. Albert Einstein 500, Campinas, SP 13083-852 Brazil; 3 Department of Chemistry, Laboratory of Fundamental and Applied Electrochemistry, Federal University of Jequitinhonha E Mucuri’s Valley, Rodovia MGT 367, km 583, 5000, Alto da Jacuba, Diamantina, MG 39100-000 Brazil

**Keywords:** Supercapacitors, Electrochemistry

## Abstract

It is common to find in the literature different values for the working voltage window (WVW) range for aqueous-based supercapacitors. In many cases, even with the best intentions of the widening the operating voltage window, the measured current using the cyclic voltammetry (CV) technique includes a significant contribution from the irreversible Faradaic reactions involved in the water-splitting process, masked by fast scan rates. Sometimes even using low scan rates is hard to determine precisely the correct WVW of the aqueous-based electrochemical capacitor. In this sense, we discuss here the best practices to determine the WVW for capacitive current in an absence of water splitting using complementary techniques such as CV, chronoamperometry (CA), and the electrochemical impedance spectroscopy (EIS). To accomplish this end, we prepare and present a *model system* composed of multiwalled carbon nanotubes buckypaper electrodes housed in the symmetric coin cell and soaked with an aqueous-based electrolyte. The system electrochemical characteristics are carefully evaluated during the progressive enlargement of the cell voltage window. The presence of residual Faradaic current is verified in the transients from the CA study, as well as the impedance changes revealed by EIS as a function of the applied voltage, is discussed. We verify that an apparent voltage window of 2.0 V determined using the CV technique is drastically decreased to 1.2 V after a close inspection of the CA findings used to discriminate the presence of a parasitic Faradaic process. Some orientations are presented to instigate the establishment in the literature of some good scientific practices concerned with the reliable characterization of supercapacitors.

## Introduction

Electrochemical capacitors (ECs) or supercapacitors (SCs) have attracted the attention of the scientific community due to their unique features related to high specific power (*P*), moderate specific energy (*E*), and long lifespan (e.g., > 100,000 charge–discharge cycles)^1,2^. To increase the *E*-values, we can focus on the capacitance and/or the *working voltage window* (WVW) or *practical capacitive voltage window* (PCVW) since *E* = *CU*^2^/2, where *U* is the cell voltage and *C* is the specific capacitance. Both *C* and *U* parameters are affected by the nature of the electrolyte and electrode materials. The most used electrolytes in ECs are organic-based since the latter enable operational conditions up to ~ 3 V^3^. However, organic electrolytes are toxic, flammable, challenging to handle because they are susceptible to contamination with air humidity, and quite expensive^4^. On the contrary, aqueous-based electrolytes are not costly, and they are highly conductive, easy to handle, non-flammable, and environmentally friendly. That is why the aqueous-based electrolytes have attracted so much attention recently for applications in ECs^5^.

The main disadvantage exhibited by aqueous electrolytes is the low PCVW predicted in as a result of the water-splitting reaction. In this sense, there is great confusion in the literature regarding the PCVW-values that can be effectively achieved in the case of the aqueous-based supercapacitors. In our opinion, the discrepancies verified in the literature are mainly related to the fact that erroneous assumptions are commonly made considering only the *electrostatic character* of the *charge-storage process* occurring in the *electrical double-layer structure*, i.e., the physicochemical aspects involving the electrolyte stability and the water-splitting process are commonly disregarded by several authors. Hypothetically, we might consider from a *purely electrostatic viewpoint* that a *single symmetric supercapacitor* (e.g., a two-electrode cell system) can be adequately described as a passive system composed of *two identical capacitors connected in series* due to the existence of *two electrical double-layer in sequence*. However, the *maximum cell voltage*, where the water is chemically stable, thus resulting in the existence of a PCVW where the electrical-double layer behavior dominates the electric response, is dictated by thermodynamic and kinetic considerations, since the electrochemical cell can become a ‘reactive system’ losing its apparently ‘passive character’ when a voltage threshold is achieved permitting the occurrence of the water electrolysis. As a result, we might erroneously consider that under *standard thermodynamic conditions* (please, see further discussion) a conventional symmetric coin cell would exhibit a *minimum cell voltage* (*U*_min_) value of 2.46 V^6^. However, this is not the case since in practice the standard (idealized) thermodynamic value of 1.23 V for the virtual chemical equilibrium involving the irreversible water-splitting process is indeed composed by two different half-reactions occurring at the positive (anode) and negative (cathode) electrodes^5, 7^.

According to the principles of chemical thermodynamics, when the activity coefficients (*a*) and fugacities (*f*) of the relevant chemical species are considered unitary (e.g., the standard conditions), we have that a *minimum cell voltage* (*U*_min_) of 1.23 V, measured under *zero-current (equilibrium) conditions*, must be applied to establish a *hypothetical chemical equilibrium* for the water electrolysis, which is, in reality, an intrinsically irreversible reaction^5^. Considering the two *half-reactions* occurring simultaneously at the anode(+) and the cathode (−) to originate the water electrolysis, we have that (please, see ref.^7^): Δ*E*^o^(V) = *E*^*o*^_(+)_ − *E*^*o*^_(−)_ ≡ *U*_min_(V) = 1.23 + 0.0147log[*p*(O_2_)/1.0 bar] + 0.0295log[*p*(H_2_)/1.0 bar]. Firstly, we verify that the solution pH does not affect the *U*_min_ values. By contrast, one has that *U*_min_ < 1.23 V when the partial pressures *p*(H_2_) and *p*(O_2_) are lower than 1.0 bar. In practice, since finite *U*_min_-values are verified for the coin cells, we have that the partial pressures at the onset of the electrolysis must resides in the range of 0 < *p* < 1, thus resulting in a PCVW lower than 1.23 V. This outcome can be the common situation for the aqueous-based supercapacitors since pure hydrogen and oxygen gases at higher partial pressures (*p* ≅ 1 bar) are not commonly injected inside the supercapacitor prototypes (e.g., coin cells)^5^. Besides, it is worth noting when the partial pressures are *considered to be zero* at the onset of the water-splitting reaction, due to the absence of the gas species, that *U*_min_ no longer can be thermodynamically described/predicted, i.e., it becomes an undefined quantity. This is the real reason for the *arbitrary choice* of the standard conditions where *p*(H_2_) = *p*(O_2_) ≡ 1 are used to ‘obtain (predict)’ the theoretical value of Δ*E*^o^ = *U*_min_ = 1.23 V.

Despite the above thermodynamic (equilibrium) considerations, we have in practice for a given electric current (*I*) (e.g., kinetic conditions) flowing in the electrochemical system that the *overall cell voltage* (*U*) for the water electrolysis is given by *U* = Δ*E*^o^ + *η*_(OER)_ + *η*_(HER)_ + *IR*_ESR_, where *η*_(OER)_ and *η*_(HER)_ are the *overpotentials* for the gas-evolving reactions, and the *IR*_ESR_ is the ohmic drop due to the presence of an equivalent series resistance (ESR). Therefore, we can argue that the different PCVWs commonly verified in the literature for several different aqueous-based electrolytes, and electrode materials, are mainly due to the different electrocatalytic activities exhibited by the anode and cathode materials for the OER and HER. As can be seen, the question regarding the theoretical predictions of the PCVW is not straightforward since it involves several chemical and thermodynamic aspects.

From the above considerations, we must emphasize that very high working voltage ranges (e.g., ~ 2.0 V) in aqueous media can be achieved in practice using the *water-in-salt electrolytes* (WISEs)^8–10^. In this case, the interface and bulk properties of the solvent can be drastically affected due to an excess of salt particles in relation to the water molecules which in turns lead to very strong short-range coulombic interactions, i.e., pronounced deviations from the ideal solution behavior can be expected for WISEs since the *activity* and *osmotic coefficients* are affected by the strong solute–solute interactions. As a result, the relative permittivity (*ε*_r_) and the specific conductance (*δ*) inside the cell can be drastically affected thus altering the specific capacitance and the electrolyte resistance, as well as the overpotential for the gas-evolving reactions due to water-splitting^11–13^. The latter effect is the major factor to obtain in practice a high capacitive working voltage range in an aqueous medium.

Several literature reports discussed that aqueous-based supercapacitors can exhibit voltage values higher than 1.23 V^14–18^. Some works even reported values of up ~ 1.5 V to ~ 3.0 V^16–31^. As can be verified, the question regarding the practical capacitive voltage ranges attained for different aqueous-based electrolytes is a source of great confusion in the literature. Therefore, several research groups, including ourselves, are devoting efforts to optimize the electrolyte conditions to improve the practical (experimental) working voltage window for aqueous-based supercapacitors. It is worth mentioning that electrochemical methods such as cyclic voltammetry (CV) and galvanostatic charge–discharge (GCD) are not very sensitive to detect the water-splitting process since the use of a dynamic variable (e.g., the scan rate or the applied gravimetric current) masks the occurrence of the irreversible ‘parasitic’ electrochemical reactions. As a result, only after the long-term GCD we can indirectly detect the gas evolution process through the internal pressure increase. On the contrary, with the use of the *single-pulse chronoamperometr*y (CA) and/or the *electrochemical impedance spectroscopy* (EIS) techniques the *real capacitive voltage interval* where the electrolyte decomposition is absent can be detected.

In the case of CA, we have after application of the different voltage pulses that the nature of the measured current can be easily discriminated, i.e., a true capacitive current must exponentially decrease to zero while in the case of the Faradaic current a non-negligible constant current is verified after longer polarization times. Considering the EIS experiments, the occurrence of a true capacitive process in the presence of an ESR is characterized by an inclined line in the complex-plane (Nyquist) plot that is almost parallel to the imaginary axis due to occurrence of a phase angle (*ϕ*) very close to − 90°. On the contrary, the presence of a Faradaic current implies in the existence of a *charge-transfer resistance* (*R*_ct_) in parallel to the *electrical double-layer capacitive behavior* and, therefore, the complex-plane plot exhibits a well-defined *depressed semicircle* whose radius progressively decreases as a function of the applied voltage.

From the above considerations, the aim of the present work is the application of different electrochemical techniques to correctly identify the true *working voltage window* (WVW) for aqueous-based supercapacitors. We hope that our work aids different researchers to identify true capacitive voltage intervals thus avoiding the publication of illusory/erroneous specific capacitance, energy, and power for supercapacitors due to the undesirable presence of a parasitic Faradaic process.

## Experimental details

We applied an all-carbon electrode to be an electrochemical platform for study this work. For that, we prepared buckypaper (BP), combining multi-walled carbon nanotubes (MWCNTs) with activated carbon (AC) on ratio mass of 1 (MWCNT):2 (AC), which is our optimized version we produced in our lab. MWCNTs were purchased from CNT Co., Ltd., Korea (conditions: Ctube-120, 95% purity, diameter from 10 to 40 nm, and length from 1 to 25 μm). AC was purchased from Kuraray Co., Ltd, Japan (conditions: YP-50F, surface area 1692 m^2^ g^−1^, bulk density 0.3 g mL^−1^). Sodium dodecyl sulfate (SDS) was purchased from Synth, 90% purity. Purum P.A. products were used throughout.

We sonicated 200 mL of deionized water with SDS in the presence of 25 mg of MWCNTs and 50 mg of AC for 30 min (constant duty-cycle, 80 W)^32^. The suspension was then slowly filtered using vacuum filtration with a 0.22 μm PTFE membrane from Millipore. The entire process was accomplished after 2 h. Samples were washed several times with deionized water to remove all SDS. Buckypaper was peeled directly from the PTFE membranes and dried at room temperature overnight. The BP was cut with a diameter of 1.2 cm to fit the CR2032 coin cell.

Scanning electron microscopy (SEM) experiments were performed using a FEI inspect F50 microscope. Raman spectrum was taken using a Renishaw inVia Raman spectroscope employing a 633 nm excitation wavelength.

All electrochemical analyses were performed using CR2032 coin cell, two identical optimized BP discs as the electrodes, a porous cellulosic membrane separator soaked in 1.0 M Li_2_SO_4_ aqueous solution. Electrochemical studies started after the activation process accomplished by applying twenty consecutive voltammetric cycles to permit adequate electrolyte penetration inside the porous electrode structure. For this first stability test, we ranged the cell voltage from 0 to 0.7 V, where we have only the electrostatic process. For the first window, we increased the voltage from 0 to 0.1 V, and then back to 0 V. In the sequence, we increased from 0 to 0.2 V and then back to 0 V, and so on. For each window, we performed CV, CA, and EIS experiments before extending the voltage to the next level.

For each voltage window, voltammetric curves (VCs) were registered using the scan rates of 1, 10, and 100 mV s^−1^. To assure internal consistency regarding the different electrochemical findings, CA experiments were performed by applying a voltage step function where the latter was fixed equal to the maximum (vertex) voltage previously verified in the voltammetric experiments. For the different windows, the transient current was measured during 300 s. Accordingly, for each voltage window, an EIS experiment was accomplished using a d.c. bias equals the vertex voltage verified in the voltammetric analysis. A frequency range of 100 kHz to 10 mHz with ten points collected per decade was used in all cases by applying a small sinusoidal perturbation of 10 mV (peak-to-peak) to ensure linearity on the impedance response. Electrochemical studies were performed using a model VersaSTAT 4 potentiostat from Ametek.

## Results and discussion

**Figure 1:**
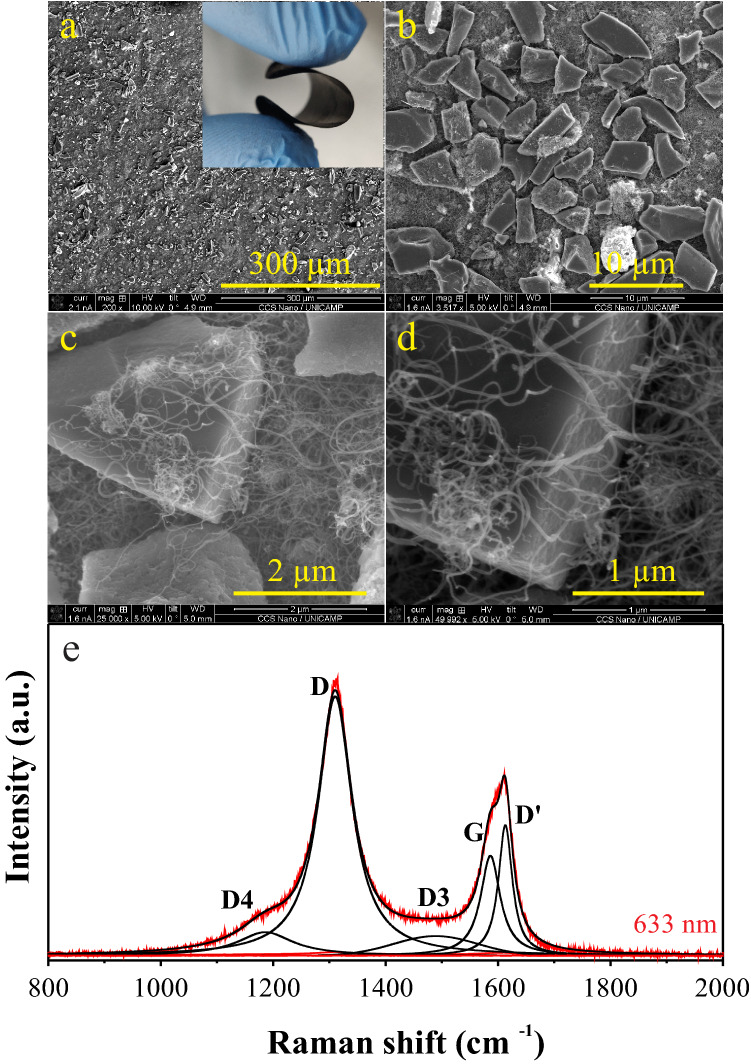
SEM micrographs (**a**–**d**) and (**e**) Raman spectrum took from BP electrodes. The inset in (**a**) shows a photo of the as-prepared flexible BP disc electrode used in the CR2032 coin cell.

Figure 1 presents typical SEM micrographs (a–d) of the bulkypaper (BP) electrodes used in this work. The inset of Fig. 1a shows a picture of the as-prepared flexible BP disc electrode. From SEM data, we can observe a highly porous electrode surface composed of ~ 7-µm AC particles and MWCNTs with an average diameter of ~ 30 nm, and length higher than 2 µm. The entangled MWCNTs interconnect several AC particles forming a complex porous network. The connections between CNTs and ACs allowed for greater mechanical strength and flexibility, as well as higher electronic conductivity. The obtained carbon electrode is robust and easy to handle.

**Figure 2:**
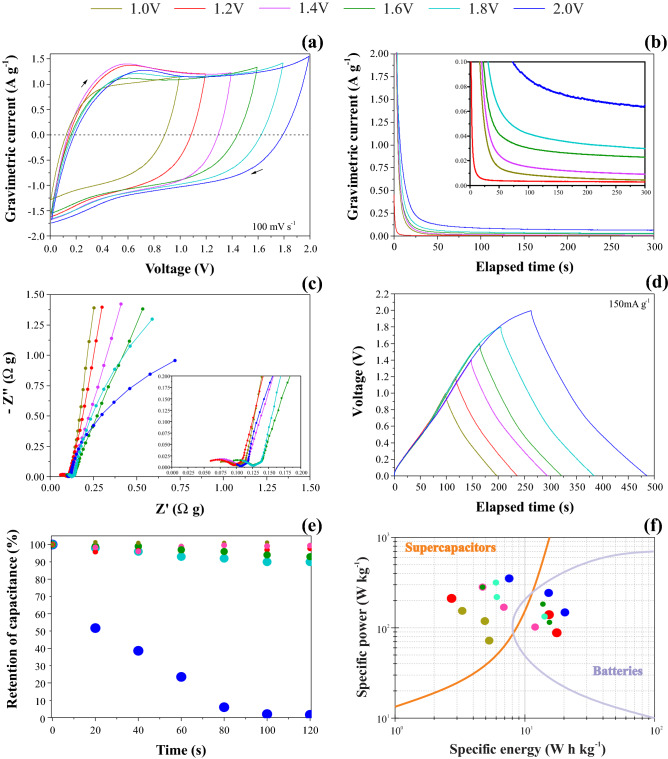
Electrochemical findings obtained for the symmetric CR2032 supercapacitor prototype containing a 1.0 M Li_2_SO_4_ solution: (**a**) voltammetric curves with different working voltage windows**;** (**b**) chronoamperometric data; (**c**) impedance findings (Nyquist plots); (**d**) galvanostatic charge–discharge curves; (**e**) cyclability test, and (**f**) Ragone Plot.

Figure 2e shows the Raman spectrum for the as-prepared BP sample which we deconvoluted into five peaks (e.g., four D-like peaks and one G-peak). The D-peak appears when the lattice vibration (Raman-inactive process) hits a defect causing the breaking of the symmetry. Accordingly, the D-peak is double resonant LO phonons around the K point^33^ while the G-band is an E_2g_ optical mode centered at 1582 cm^−1^, originated from the double degenerate vibrational mode of iLO and iTO phonon branches crossing at the Γ point in the first Brillouin zone of the graphite structure. The G-peak corresponds to the high-frequency E_2g_ phonon at Γ and D is the breathing modes of six-atom rings, requiring a defect for its activation^34^.

Figure 2 shows several electrochemical findings obtained using different experimental techniques. Firstly, the criterion to register the CVs in a given electrolyte using a potentiostat is the accurate choice of a voltage window where the electrolyte is stable and the electrodes do not undergo wear. As a result, we expect to obtain almost rectangular (“mirror-like”) voltammetric profiles. For sake of running a large number of assisted experiments extending the voltage window, it is more pleasured to scan cells at relatively fast scan rates (e.g., ~ 100 mV s^−1^), which is also more close to fast charge/discharge events occurring in the supercapacitor. However, for this relatively high scan rates, a small parasitic Faradaic current is present due to water electrolysis. This is the normal case even when we have quasi-rectangular EDLC patterns for CVs since the cyclic voltammetry is not sensitive to discriminate a pure capacitive process from an irreversible gas-evolving reaction occurring in parallel with a relatively small current efficiency.

**Figure 3:**
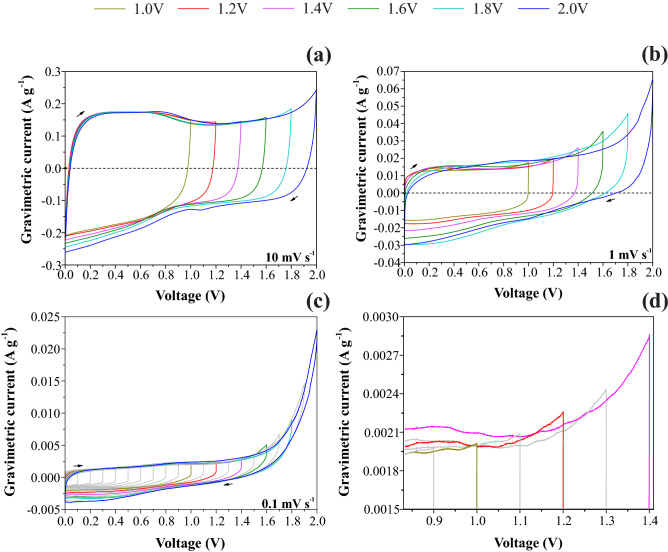
Voltammograms obtained at (**a**) 10 mV s^−1^, (**b**) 1 mV s^−1^, (**c**) 0.1 mV s^−1^ and (**d**) zoom in the region 0.9 to 1.4 V of the graphic (**c**) for the symmetric coin using BP electrodes soaked with a 1.0 M Li_2_SO_4_ aqueous solution.

Figure 3a shows CVs obtained at 100 mV s^−1^ as a function of the anodic vertex voltage, which behavior is characteristic of supercapacitors. It is worth mentioning that the presence of a ’tail’ at the vertex voltage is commonly adopted as clear evidence of the water-splitting process. However, this criterion alone is not entirely satisfactory for practical purposes. Please, from Fig. 3, see a further discussion regarding the crucial influence of the scan rate on the CV profiles.

From the above considerations, we performed additional experiments using the CA technique to correctly identify the eventual presence of a Faradaic current occurring during the transient current characteristic of a capacitive discharge process. After a long polarization time necessary for a practical full discharge of the device in the absence of a Faradaic (parasitic) current (see Fig. 2b), we observed for voltages higher than 1 V the presence of almost stationary currents whose magnitude dramatically increased with the window voltage (e.g., 2.6 mA g^−1^ @ 1.2 V and 64 mA g^−1^ @ 2 V). These findings confirmed the occurrence of the water-splitting reaction, which was not possible to detect using only the CV technique. Therefore, we can affirm that our aqueous-based EDLC device can only be operated with confidence in the absence of parasitic gas-evolving reactions when *U* ≤ 1.2 V.

Even considering that the CA findings are quite conclusive to correctly identify the real capacitive voltage window for supercapacitors, we performed further complementary studies in the frequency domain using the EIS technique. Figure 2c shows the Nyquist plots obtained for different d.c. bias corresponding to the voltages used in the CV and CA experiments. For lower d.c. voltages (e.g., 1 and 1.2 V), it was verified in the low-frequency range of the spectra a strong capacitive behavior characterized by an exponent (*n*) of the constant phase element (CPE) higher than 0.9. Considering that *Z*_CPE_ = 1/*Y*_0_(*jω*)^*n*^, when *n* = 1 one has that *Y*_0_ = *C*_edl_, i.e., the electrical double-layer capacitance is unambiguously determined. However, due to the dispersive capacitive effects, inherent to the presence of surface inhomogeneities in solid electrodes, using the well-known Brugg–Sluyters formula we have as a guide for the precise determination of the capacitance value the practical interval of 0.9 ≤ *n* ≤ 1. For these conditions regarding the CPE behavior, we have that the impedance response in the low-frequency range for EDLCs is characterized by a straight line almost parallel to the imaginary (Z^//^) axis. As seen in Fig. 2c, for higher voltages (e.g., *U* ≥ 1.4 V) the EIS findings at low frequencies start to form a semicircle characterized by *n* < 0.9 since in these cases we have strong deviations from the ideal capacitive behavior due to the presence of a Faradaic component in the Nyquist plot. The inset of Fig. 2c evidences the medium–high frequency region where the capacitance dispersion can be attributed to the well-known porous electrode behavior. More precisely, for this type of dispersive effect where we identify the presence of a high-frequency semicircle followed by an inclined line at intermediate frequencies with a phase angle close to − 45°, the porous electrode behavior can be represented by a two-channel transmission line incorporating the anomalous (non-Fickian) transport of the charge carriers in the interconnected solid and liquid phases, i.e., in this case, the Bisquert #2 impedance model can be adequately used for simulation of the impedance data using, for example, the Zview and NOVA software. From these considerations, we can affirm from the perspective of the frequency domain analysis that *U* = 1.4 V is the maximum voltage window that can be used with confidence to avoid the presence of water-splitting in the coin cell.

A comparison of the CA and EIS findings reveals that the former technique is more sensitive for the quantitative identification of parasitic Faradaic reaction occurring simultaneously with the electrical double-layer charge-discharging processes. In addition, it became obvious for us that the CV technique is the worst electrochemical technique to be used when the proposal is the accurate identification of the capacitive voltage window for EDLCs.

Figure 2d shows the galvanostatic charge–discharge (GCD) curves obtained for the different voltage windows used to determine the coulombic efficiency and the ESR value. Electrochemical data obtained from GCD analysis were gathered in Table 1. As can be seen, the coulombic efficiency (CE) reduced with the increasing voltage window, i.e., unacceptable values were observed above 1.6 V.

**Table 1:** CE and ESR values as a function of the working voltage window (WVW).

WVW (V)	CE/%	ESR/Ω g
1.0	100	0.16
1.2	100	0.16
1.4	99	0.18
1.6	96	0.17
1.8	88	0.18
2.0	85	0.20

As seen, the CE values only evidenced the presence of a parasitic Faradaic process when *U* ≥ 1.4 V. Besides, we verified that the GCD curves have similar triangular profiles characteristic of well-behaved supercapacitors even considering the presence of a small parasitic Faradaic process at voltages higher than 1.2 V (see the previous discussion). Again, we can affirm that the use of the GCD method alone is not satisfactory for the correct identification of the capacitive voltage window. From the GCD findings, we determined the ESR values using the abrupt voltage drop (*ESR* = Δ*U*_drop_/2*I*)^35^. As seen in Table 1, the ESR values slightly increased as a function of the cell voltage. In principle, this behavior can be ascribed to the presence of gas bubbles from the water-splitting reaction inside the coin cell, which in turn increased the overall cell resistance.

The practical consequences of the inadequate choice of the voltage window for supercapacitors are shown in Fig. 2e. As seen, there was an exponential decay of the capacitance value to zero after a cycling process using the floating time method accomplished during 120 min at 2.0 V. On the contrary, there was almost no impact (e.g., 96%) on the capacitance retention at 1.2 V. The accumulation of gases inside the hermetically sealed coin cell provided by the water-splitting process was so great that it caused irreversible damage for the coin cell operated at 2.0 V (i.e., it expanded to open up). Unfortunately, some researchers are inclined to push the working window voltage to erroneously suggest that their cells exhibit more energy and power than other systems reported in the literature. This type of biased (incorrect) characterization of supercapacitors can lead to illusory figures-of-merit as can be seen in Fig. 2f. For instance, the Ragone plot indicated that at 2.0 V the cell has higher energy and power storage capabilities, which is not true since for these conditions part of the electric current is due to water-splitting instead of the reversible charge-storage process.

In several literature reports, the CV experiments are accomplished using high scan rates (*ν* ≥ 50 mV s^−1^). In this sense, we performed complementary experiments using very low scan rates from 10 to 1.0 mV s^−1^. These complementary findings are shown in Fig. 3. From the theoretical viewpoint, a ‘perfect supercapacitor’ must exhibit a symmetric response of rectangular shape for all scan rates when the ESR is negligible. Therefore, a change in the CV profile as a function of the scan rate due to the appearance of an ‘anodic tail’ close to the vertex voltage is an indication of the occurrence of a parasitic Faradaic reaction, as is the case of the electrolyte decomposition. In this sense, we verified that the apparent capacitive voltage window decreased from ≈1.6 to ≈1.2 V when the scan rate was reduced from 10 to 0.1 mV s^−1^, respectively. On the whole, these complementary findings support the previous CA results where a true capacitive window was verified only when *U* ≤ 1.2 V.

## Conclusion

We reported in this work on the best scientific practices regarding the correct identification of the capacitive working voltage window for supercapacitors. We used as a model device a symmetric aqueous-based supercapacitor composed of carbon-based buckypaper electrodes. Our motivation for this study is the continuous verification in the literature of works that report unrealistic/incorrect figures-of-merit for supercapacitors (e.g., the specific energy and power). We demonstrated here that although there is a pronounced capacitive characteristic verified in the voltammetric study (e.g., quasi-rectangular shaped voltammograms), that these findings can mask the parasitic occurrence of the Faradaic water-splitting process. We verified that the cyclic voltammetry (CV) or galvanostatic charge–discharge (GCD) approaches cannot be used with confidence to correctly identify the capacitive working voltage window for supercapacitors. As a result, complementary studies involving the application of the chronoamperometry (CA) and the impedance (EIS) techniques are highly recommended, if not mandatory, for the accomplishment of a reliable electrochemical characterization of supercapacitors. To quote, we verified that an apparent voltage window of 2 V determined using the CV technique is drastically decreased to 1.2 V after a close inspection of the CA findings used to discriminate the presence of a parasitic Faradaic process. The results obtained with the EIS technique are in close agreement with those determined using the CA technique and in disagreement with the CV findings. In addition, while the cell remained quite stable for the charge-storage process accomplished at 1.2 V, the same cell was completely deactivated after a brief time interval when a voltage of 1.6 V was applied. We hope with this work to instigate the publication of more reliable findings regarding the electrochemical characterization of supercapacitors.
